# Supplementary material for: Exploring the Gender and Age Demographics of Patients Treated by Emergency Medical Teams during Disasters
Source: Int J Environ Res Public Health. 2024 May 28;21(6):696. doi: 10.3390/ijerph21060696 (PMC11203569; doi:10.3390/ijerph21060696)
Supplement: Supplementary file 1 [file ijerph-21-00696-s001.zip › ijerph-2952440-supplementary.pdf]

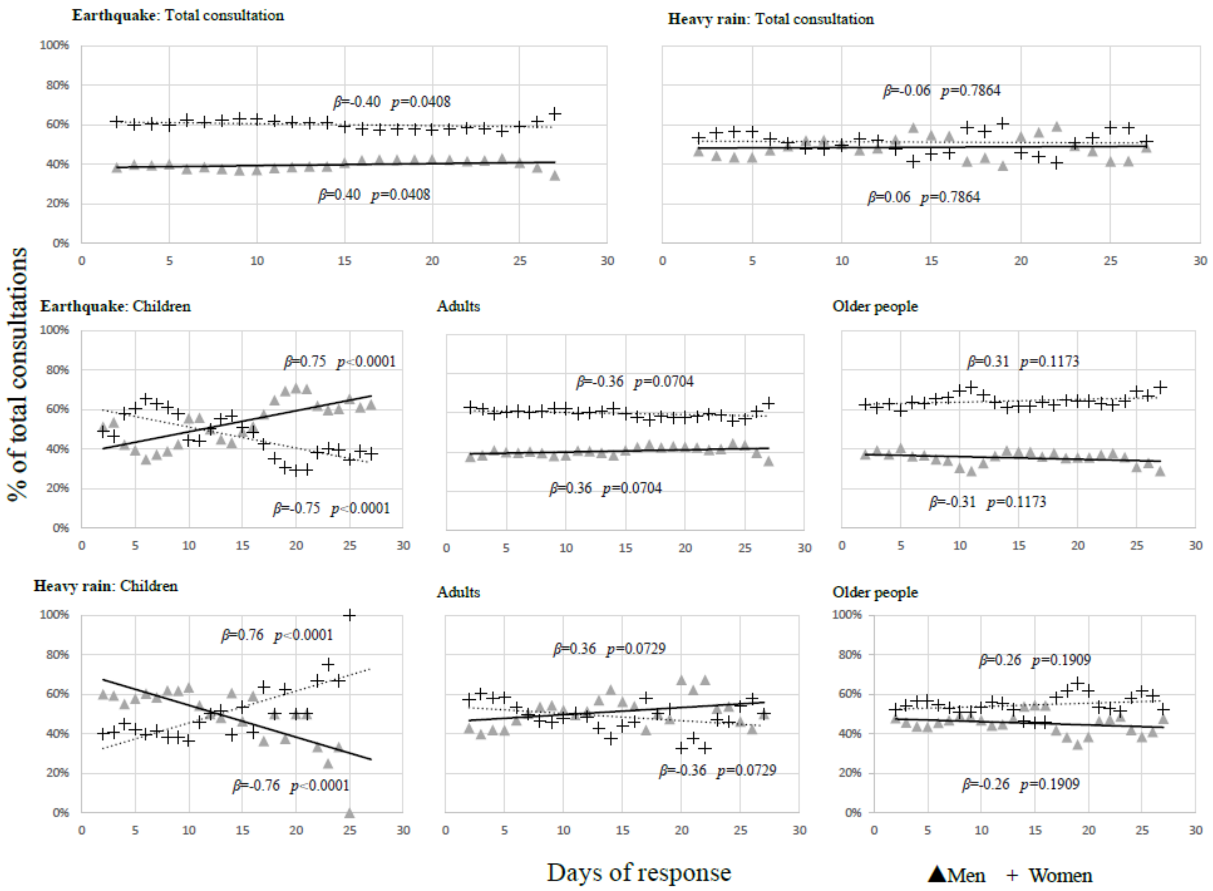

Supplementary Figure S1. Trends in medical consultations by gender in age groups: a comparison between earthquakes and heavy rain events

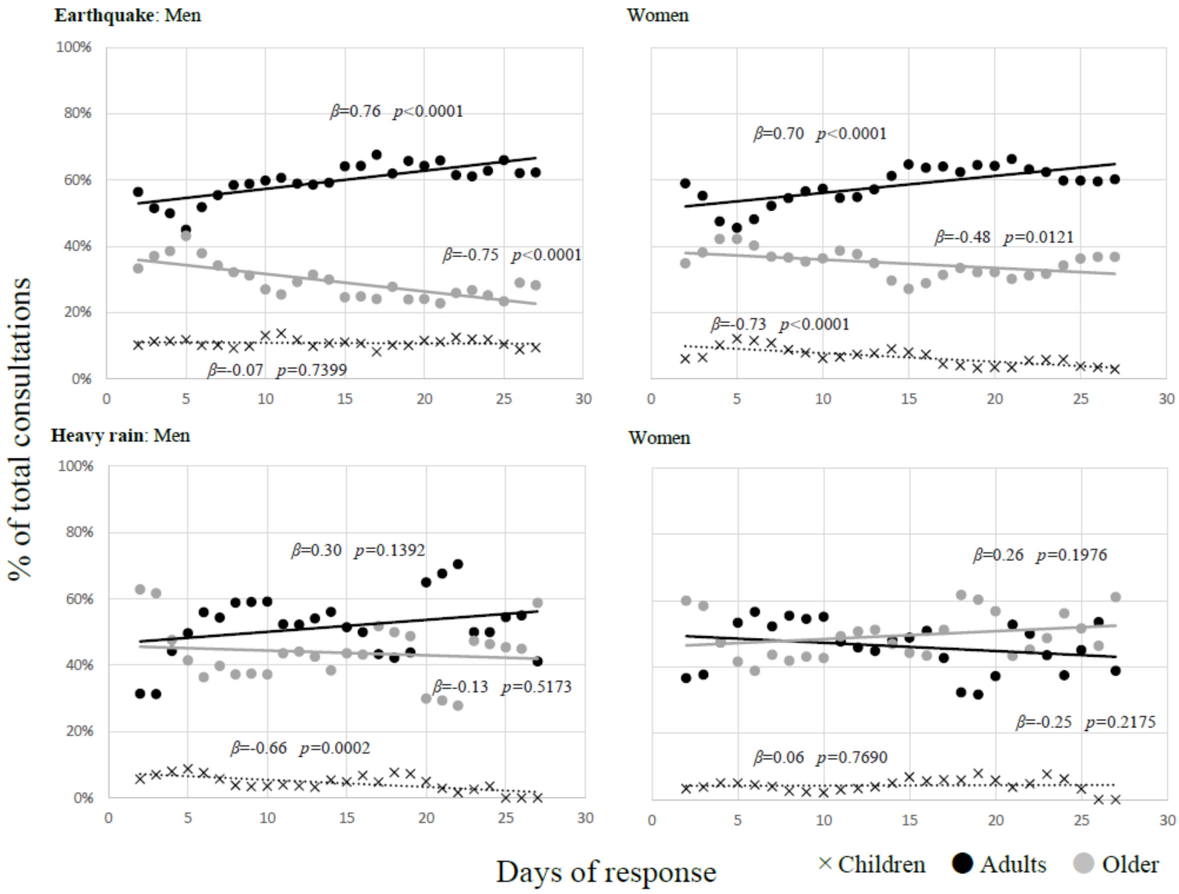

Supplementary Figure S2. Trends in medical consultations by age group in gender: a comparison between earthquakes and heavy rain events
